# Hospital Note

**Published:** 1898-03

**Authors:** 


### Hospital Note.

It was announced recently in the newspapers that a number of Buffalo physicians, including Drs. Bernhard Cohen, of Niagara street, J. N. Goltra, of Elmwood avenue, John E. Bacon, of Prospect avenue, William H. Thornton, of Niagara street, C. A. Wall, of Hudson street, F. E. Luke, of Richmond avenue, L. J. McAdam, of Jersey street and Frederick Millener, of Niagara street, met at the University Club, January 20, 1898, and organised an association to be known as the Buffalo Polyclinical Association.

Their intention in forming the association is ultimately to build another hospital in this city and the proposition was discussed by all present. A number of suggestions were offered as to the best mode of procedure in perfecting preliminary arrangements.

Drs. Goltra and Wall were appointed a committee to draft a constitution and by-laws. Other meetings will be held for the further discussion and perfecting of the plans.

